# Disintegrin targeting of an α_v_β_3_ integrin-over-expressing high-metastatic human osteosarcoma with echistatin inhibits cell proliferation, migration, invasion and adhesion *in vitro*

**DOI:** 10.18632/oncotarget.10111

**Published:** 2016-06-16

**Authors:** Yasunori Tome, Hiroaki Kimura, Tasuku Kiyuna, Naotoshi Sugimoto, Hiroyuki Tsuchiya, Fuminori Kanaya, Michael Bouvet, Robert M. Hoffman

**Affiliations:** ^1^ AntiCancer, Inc., San Diego, CA 92111, USA; ^2^ Department of Surgery, University of California, San Diego, CA 92103, USA; ^3^ Department of Orthopedic Surgery, Graduate School of Medicine, University of the Ryukyus, Okinawa, Japan 903-0125; ^4^ Department of Orthopaedic Surgery, Graduate School of Medical Science, Kanazawa University, Kanazawa, Japan 920-8641; ^5^ Department of Physiology, Graduate School of Medical Science, Kanazawa University, Kanazawa, Japan 920-8641

**Keywords:** α_v_ β_3_ integrin, echistatin, green fluorescent protein/red fluorescent protein, osteosarcoma, metastasis

## Abstract

The *in vitro* efficacy of the disintegrin echistatin was tested on a high-metastatic variant of 143B human osteosarcoma, 143B-LM4, which over-expresses α_v_β_3_ integrin. Echistatin is an RGD cyclic peptide and an antagonist of α_v_β_3_ integrin. In the present study, echistatin inhibited cell proliferation, migration, invasion, and adhesion of 143B-LM4 cells. 143B-LM4 cell proliferation decreased after treatment with echistatin in a time-dependent and dose-dependent manner (*P* <0.01). *In vitro* migration and invasion of 143B-LM4 cells were also inhibited by echistatin in a dose-dependent manner (*P* <0.01, respectively). Cell adhesion to vitronectin of 143B-LM4 cells was also inhibited by echistatin in a dose-dependent manner (*P* <0.01). These results suggest that α_v_β_3_ integrin may be an effective target for osteosarcoma.

## INTRODUCTION

Osteosarcoma is the most common bone cancer in children and adolescents [1–3]. Osteosarcoma usually arises in the distal femur and proximal tibia and proximal humerus.

Development of neo-adjuvant chemotherapy has increased the rate of overall survival and has enabled surgery, but the failure rate is still high, causing death in children and adolescents [4–7].

Novel more effective targets for osteosarcoma therapy are therefore needed. Toward this goal, α_v_β_3_ integrin-over-expressing high-metastatic variants of the human osteosarcoma cell line 143B were previously isolated and termed 143B-LM4.

Over-expression of α_v_β_3_ integrin in the variant selected for high-metastasis suggested that integrin maybe a promising target for osteosarcoma. The 143B-LM4 cells expressed green fluorescent protein (GFP) in the nucleus and red fluorescent protein (RFP) in the cytoplasm, and therefore could be imaged down to the subcellular level *in vitro* and *in vivo* [8].

Lung seeding by 143B-LM4 cells was directly imaged and found to be greatly inhibited by the anti-β1 integrin monoclonal antibody, AIIB2. AIIB2 also significantly inhibited spontaneous lung metastasis and increased survival of mice with orthotopically-growing 143B-RFP [9].

In the present study, we tested echistatin, a cyclic RGD peptide antagonist of α_v_β_3_ integrin (disintegrin) [10], as a molecular-targeting drug in human metastatic osteosarcoma *in vitro* on the highly metastatic 143B-LM4 cell line which over-expresses α_v_β_3_ integrin described above.

## RESULTS AND DISCUSSION

### Dual-color-labeled GFP- and RFP-expressing 143B-LM4 cells

**Figure 1:**
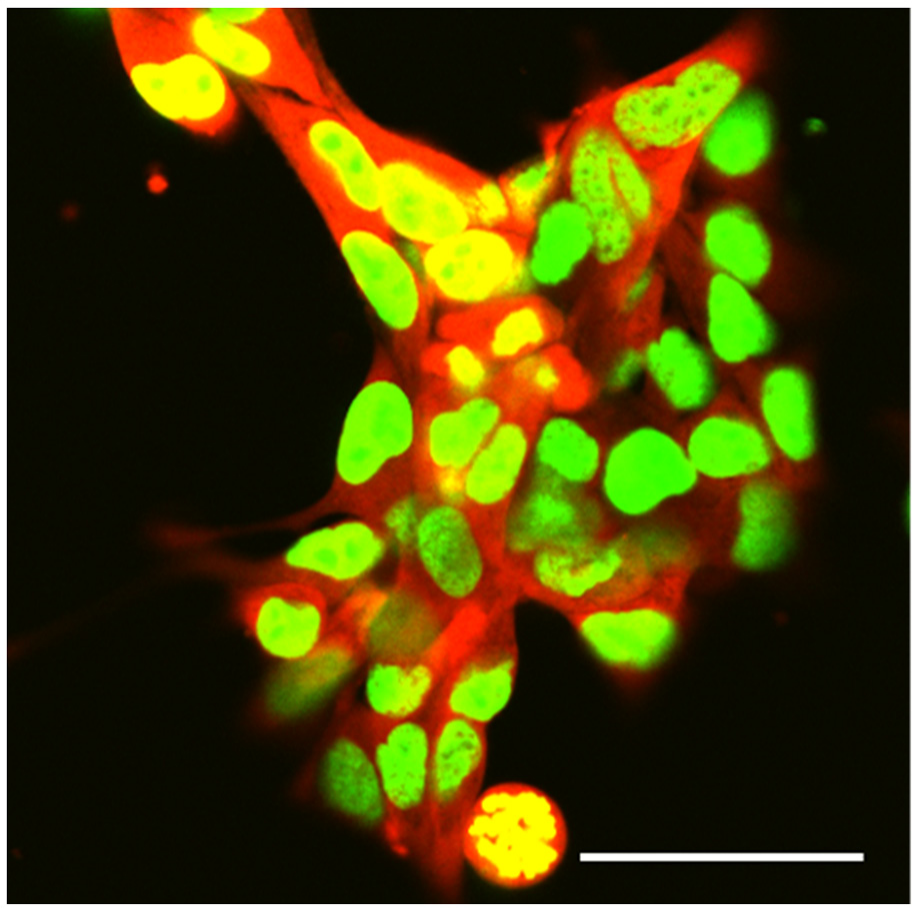
Dual-color selected 143B-LM4 human osteosarcoma cells expressing GFP in the nucleus and RFP in the cytoplasm *in vitro* Images were obtained with a Fluoview FV1000 laser-scanning confocal microscope (Olympus Corp., Tokyo, Japan). GFP was excited at 488 nm, RFP at 543nm. Magnification 60x. Scale bar: 50 μm.

The high-metastatic integrin-over-expressing 143B-LM4 cells have a strikingly bright GFP in the nucleus and RFP in the cytoplasm, *in vitro* (Figure 1).

### Echistatin inhibits cell proliferation, migration, invasion, and adhesion of 143B-LM4 cells

**Figure 2:**
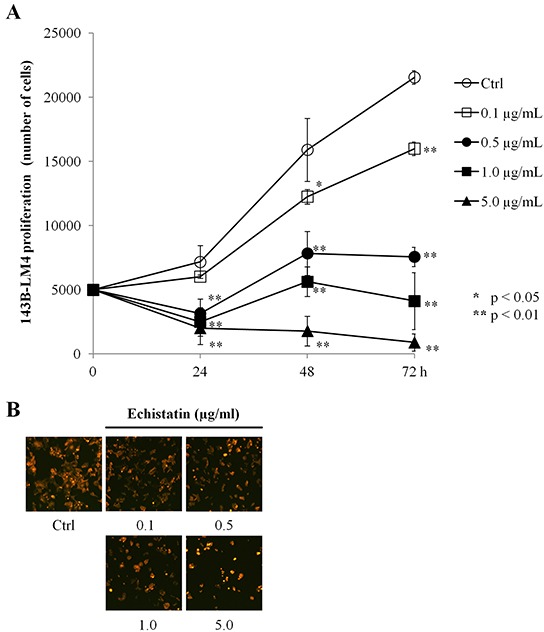
Echistatin decreased proliferation of 143B-LM4 cells *in vitro* **A.** Efficacy of echistatin on 143B-LM4 cell proliferation. Proliferation of 143B-LM4 cells was inhibited by echistatin and decreased in a time-dependent and a dose-dependent manner (*P* <0.01). *Error bars:* SEM. **B.** visualization of the efficacy of echistatin at various concentrations on 143B-LM4 cell proliferation at 72 hours. Cell number decreased in a dose-dependent manner. Images were obtained with the Olympus IX71 fluorescence microscope. Magnification, 20×.

To determine whether echistatin inhibited 143B-LM4 cell proliferation, 143B-LM4 cells were treated with echistatin at various concentrations. 143B-LM4 cell proliferation decreased after treatment with echistatin in a time-dependent and a dose-dependent manner (*P* <0.01) (Figure 2A). After 24 hr treatment, 143B-LM4 cell proliferation was decreased to 44.0% at 0.5 μg/mL; 34.8% at 1.0 μg/mL echistatin; and 28.1% at 5.0 μg/mL echistatin, compared to control (*P* <0.01, respectively). At 72 hr after treatment, cell proliferation decreased to 74.2% at 0.1 μg/mL; 35.1% at 0.5 μg/mL echistatin, 19.1% at 1.0 μg/mL; and to 4.2% at 5.0 μg/mL echistatin, compared to control (*P* <0.01, respectively). Fluorescence microscopy showed that cell number decreased in a dose-dependent manner and the cancer cells appeared more shrunken at a high concentration of echistatin (Figure 2B).

**Figure 3:**
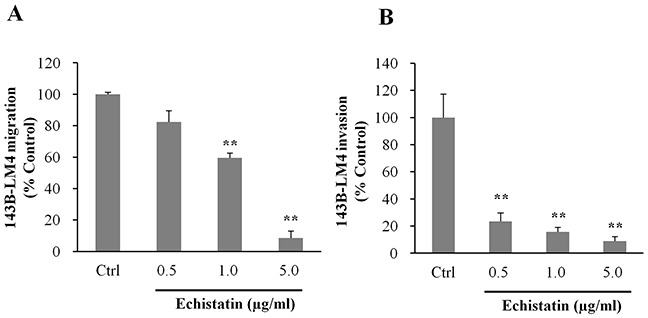
Echistatin decreased migration and invasion of 143B-LM4 cells *in vitro* **A.** Efficacy of echistatin on 143B-LM4 cell migration *in vitro*. 143B-LM4 cells were seeded into the upper compartment of transwell chambers (Corning^®^ HTS Transwell-96 uncoated plates, (Tewksbury, MA). Migration of 143B-LM4 cells decreased in a dose-dependent manner (*P* <0.01). *Error bars:* SEM. **B.** Efficacy of echistatin on 143B-LM4 invasion *in vitro*. 143B-LM4 cells were seeded into the upper compartment of transwell chambers with the surface coated with a basement membrane extract. Invasion of 143B-LM4 cells decreased in a dose-dependent manner (*P* <0.01). *Error bars*: SEM. Absorbance was evaluated with a plate reader after cells which migrated or invaded were treated with MTS.

The effect of echistatin on the ability of 143B-LM4 cells to migrate was then tested. *In vitro,* migration of 143B-LM4 cells decreased to 59.4% at 1.0 μg/mL and to 8.5% at 5.0 μg/mL echistatin, compared to control (*P* <0.01 respectively) (Figure 3A).

Invasion of 143B-LM4 cells decreased to 23.3% at 0.5 μg/mL, 15.7% at 1.0 μg/mL and to 9.0% at 5.0 μg/mL echistatin, compared to control (*P* <0.01, respectively) (Figure 3B).

**Figure 4:**
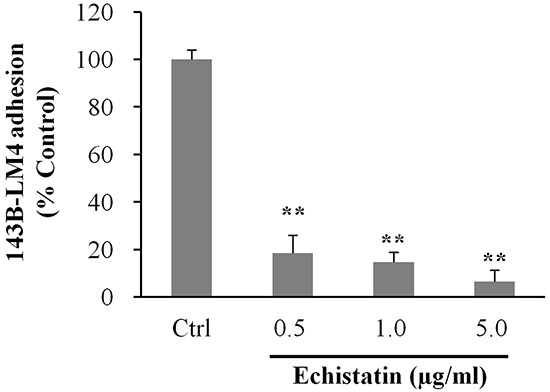
Echistatin decreased adhesion to vitronectin of 143B-LM4 cells *in vitro* Efficacy of echistatin on 143B-LM4 cell adhesion to Vitronectin (Trevigen, Gaithersburg, MD) *in vitro* (Corning^®^ HTS Transwell-96 plates (Tewksbury, MA) coated with Vitronectin. Adhesion of 143B-LM4 cells decreased in a dose-dependent manner (*P* <0.01). *Error bars:* SEM. Absorbance was evaluated with a plate reader after adherent cells were treated with MTS.

To determine whether echistatin could inhibit adhesion to vitronectin, which is a specific ligand of α_v_β_3_ integrin, 143B-LM4 cells were seeded on vitronectin coated-dishes and treated with echistatin. Adhesion to vitronectin of 143B-LM4 cells decreased to 18.5% at 0.5 μg/mL, 14.6% at 1.0 μg/mL and to 6.5% at 5.0 μg/mL echistatin, compared to control (*P* <0.01, respectively) (Figure 4).

Aggressive chemotherapy of osteosarcoma in patients with metastatic or recurrent disease, most commonly in the lung [11–13], still results in poor prognosis with less than a 20% 5-year overall survival rate [14–16]. Therefore, novel targets are needed to overcome recurrence or metastasis and to improve the disease-free survival rate.

In the present study, we demonstrated that echistatin resulted in a significant decrease of cell proliferation, migration, invasion, and adhesion of 143B-LM4 cells *in vitro*. As 143B-LM4 is a high-metastatic variant that over-expresses α_v_β_3_ integrin, these results suggest that α_v_β_3_ integrin may be an effective target for osteosarcoma that has future clinical potential.

Previously-developed concepts and strategies of highly-selective tumor targeting can take advantage of molecular targeting of tumors, including tissue-selective therapy which focuses on unique differences between normal and tumor tissues [17–22].

## MATERIALS AND METHODS

### Cells

143B-LM4 cells [8] expressing GFP in the nucleus and RFP in the cytoplasm (Figure 1) were generated, as previously described, and maintained in RPMI 1640 medium (Irvine Scientific, Santa Ana, CA) containing 15% fetal bovine serum (FBS) (Omega Scientific, San Diego, CA) and 1% penicillin/streptomycin at 37°C in 5% CO_2_.

### Drug

Echistatin [10] (Tocris Bioscience, Ellisville, MO) was diluted with distilled water and stored at −20°C.

### Cell proliferation assay

143B-LM4 cells (5×10^3^) were added to 96-well tissue culture plates and placed overnight at 37°C in a CO_2_ incubator. The cells were treated with various concentrations of echistatin. At various time intervals, 100 μl of fresh medium was replenished and 20 μl of 3-(4, 5-dimethylatiazol-2yl)-5-(3-carboxymethoxyphenyl)-2H-tetrazolium (MTS) (Promega, Madison, WI) was added to the cells. After incubation for 1 h, absorbance was measured using a microplate reader (TECAN Group Ltd., Männedorf, Switzerland) at 490 nm. The assay was performed in triplicate and at least twice. Cell images were observed with a fluorescence microscope (IX71, Olympus Corp., Tokyo, Japan).

### Migration and invasion assay

An *in vitro* migratory/invasiveness assay was carried out with Corning^®^ (Tewksbury, MA) HTS Transwell-96 plates uncoated or coated, respectively, with a basement membrane extract (Trevigen, Gaithersburg, MD) according to manufacturer's instructions. 143B-LM4 cells (5×10^4^) were added to the upper chamber and various concentrations of echistatin were added to the lower chamber (0.5 μg/mL, 1.0 μg/mL, 5.0 μg/m), for both the migration and invasion assays. The lower chamber had the same conditions for the migration and invasion assays. For the migration assay, an uncoated well was used for the upper chamber. For the invasion assay, a well coated with a basement membrane was used as the upper chamber. For both assays, cancer cells were seeded in the upper chamber. The plate was placed for 24 h at 37°C in a tissue culture incubator. After incubation for 24 h, 100 μl of fresh medium was gently replenished in the lower chamber and 20 μl MTS was added to the lower chamber to determine cell viability. After incubation for 1 h, the absorbance was measured using a microplate reader at 490 nm. The assays were performed in triplicate and at least twice, independently.

### Adhesion assay

The adhesion assay was carried out with CultureCoat^®^ Vitronectin 96-well dishes (Trevigen) according to the manufacturer's instructions. 143B-LM4 cells were labeled with 2 μM calcein AM (Invitrogen, Carlsbad, CA), harvested and then re-suspended in medium to a final concentration of 1.5×10^5^ cells/ml. Only live cells can absorb this agent. 143B-LM4 cells (1.5×10^4^/100 μl) were added to each well and were either left untreated or treated with echistatin (0.5 μg/mL, 1.0 μg/mL, 5.0 μg/mL), and placed for 1 h at 37°C in a CO_2_ incubator. The medium was gently removed. The wells were washed twice gently with PBS. The fluorescence intensity of the cells remaining on the bottom of the well was measured using a SPECTRAmax GEMINI Dual-Scanning Microplate Spectrofluorometer, (Molecular Devices, Sunnyvale, CA) at an excitation/emission wavelength of 485/520 nm. The assay was performed in triplicate and at least twice independently.

### Microscopy

*In vitro* 143B-LM4 cells were imaged with a Fluoview FV1000 laser-scanning confocal microscope (Olympus Corp., Tokyo, Japan) with a XLUMPLFL 60xW (0.90 NA) water-immersion objective [23]. GFP was excited at 488 nm, RFP at 543 nm. In the proliferation assay, cells were imaged with a fluorescence microscope (IX71, Olympus Corp., Tokyo, Japan).

### Statistical analysis

The data are presented as mean ± SD or mean ± SEM. Statistical analysis was with ANOVA for multiple data sets. *P*-values of less than 0.05 were considered significant.
